# Immune checkpoint inhibitor-induced hypothyroidism predicts treatment response in Japanese subjects

**DOI:** 10.3389/fendo.2023.1221723

**Published:** 2023-07-31

**Authors:** Yuichiro Iwamoto, Tomohiko Kimura, Kazunori Dan, Mana Ohnishi, Haruka Takenouchi, Hideyuki Iwamoto, Junpei Sanada, Yoshiro Fushimi, Yukino Katakura, Masashi Shimoda, Shuhei Nakanishi, Tomoatsu Mune, Kohei Kaku, Hideaki Kaneto

**Affiliations:** Division of Diabetes, Metabolism and Endocrinology, Kawasaki Medical School, Kurashiki, Japan

**Keywords:** immune-related adverse events, immune check point inhibitors, hypothyroidism, transient thyrotoxicosis, retrospective study

## Abstract

**Background:**

Immune checkpoint inhibitors (ICIs) cause a variety of immune-related adverse events (irAEs). Among them, thyroid dysfunction is most frequently observed. Patients with irAEs have higher survival rates than those without irAEs, but there is no certainty as to whether the degree of thyroid dysfunction is associated with treatment response or survival with ICIs.

**Method:**

This is a single-center, retrospective, observational study. The study included 466 patients who received ICI at Kawasaki Medical School Hospital from September 1, 2014, to May 31, 2022 and evaluated the degree of abnormal thyroid function and survival and remission rates after treatment with ICIs. Primary hypothyroidism of less than 10 μIU/mL TSH was classified as grade 1, and primary hypothyroidism requiring more than 10 μIU/mL TSH or levothyroxine as grade 2-4.

**Result:**

The mean age of the study participants was 68.2 ± 10.3 years, and the percentage of male participants was 72.6%. The frequency of ICI-induced thyroid dysfunction in the study participants was 28.2%. TSH levels were significantly higher in Grade 1 and Grades 2-4 when treated with ICI compared to NTF (p<0.0001). The survival rate at 1 year after ICI administration was significantly higher with 64.9% for grade 1 and 88.9% for grades 2-4 compared to 52.1% for NTF (p<0.0001). Cancer stage at the time of ICI administration did not differ among the groups (p=0.68). Nevertheless, the remission rate assessed by RECIST criteria was significantly higher in grades 2-4 compared to NTF (p<0.0001).

**Conclusion:**

ICI-induced thyroid dysfunction was significantly correlated with survival, mean observation time, and treatment remission rate. It is important to monitor thyroid hormone levels regularly in patients receiving ICIs.

## Introduction

Immune checkpoint inhibitors (ICIs) cause a variety of immune-related adverse events (irAEs). Among them, thyroid dysfunction is most frequently observed in the endocrine setting (1). In one cohort study, 44% of ICI-treated patients developed some form of hypothyroidism, and most ICI-induced thyroid dysfunction was destructive thyroiditis or hypothyroidism (2). The frequency of ICI-induced Graves’ disease is low; about 2% of patients show thyrotoxicosis after ICI administration (3). In a large cohort study of patients with malignant melanoma, patients who developed thyrotoxicosis after receiving ICI showed progression-free survival, but there was no correlation between cancer outcome and hypothyroidism (4).

We conducted a single-center retrospective study of 466 patients treated with ICI at Kawasaki Medical School Hospital and reported a significantly higher survival rate in patients diagnosed with endocrine-related irAE (5). The incidence of endocrine-related irAE at our institution was 25.5%, most of which were primary hypothyroidism. In Japanese patients, the mean observation period may be longer in cases accompanied by endocrine-related irAE, but previous studies were insufficient to evaluate the correlation between the prevalence of irAE and the efficacy of ICI treatment. The purpose of this study was to evaluate the correlation between the degree of thyroid dysfunction and the efficacy of ICI therapy in ICI-treated patients whose thyroid function had been evaluated.

## Material and methods

### Study population and patient preparation

**Figure 1:**
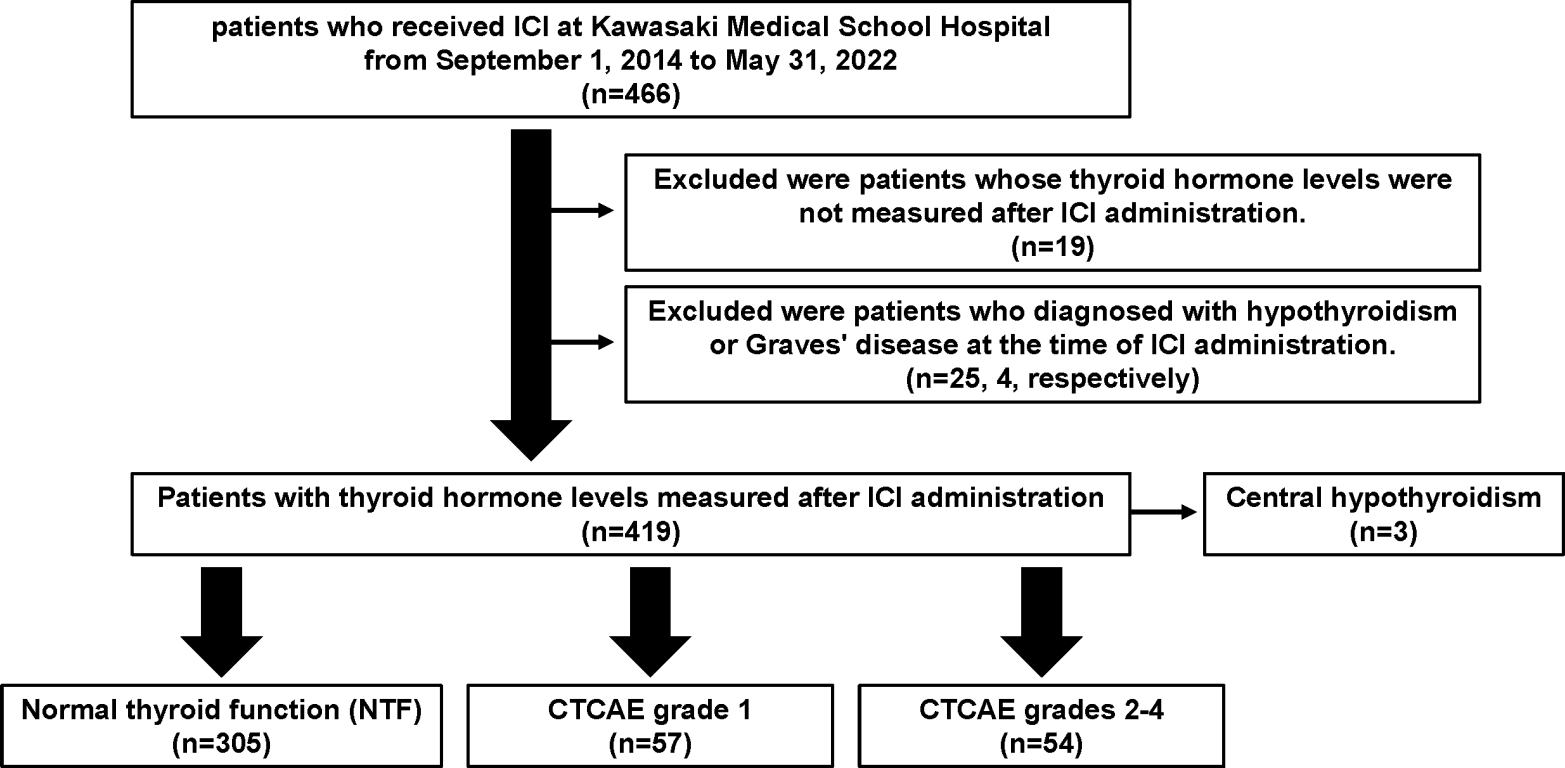
A flowchart of the participants in this study.

ICIs used in our clinic are cytotoxic T lymphocyte antigen-4 (CTLA-4) inhibitors (ipilimumab), programmed cell death protein 1 (PD-1) inhibitors (nivolumab and pembrolizumab), and programmed cell death protein 1 ligand 1 (PD-L1) inhibitors (atezolizumab, durubumab, and avelumab). This is a single-center, retrospective, observational study of a total of 466 patients who were treated with ICI at Kawasaki Medical School Hospital from September 1, 2014, to May 31, 2022. Details are as previously described (4). The study protocol, including opt-out informed consent, was approved by the Institutional Review Board of Kawasaki Medical School (No. 5726-00). The study was conducted by the principles of the Declaration of Helsinki. A flowchart of the participants in this study is shown in **Figure 1**. Of the 466 patients who were treated with ICIs at our hospital during this period, 19 patients whose thyroid hormone levels were not measured after ICI administration and 25 and 4 patients diagnosed with hypothyroidism or Graves’ disease, respectively, at the time of ICI administration were excluded from the study. According to Common Terminology Criteria for Adverse Events (CTCAE) version 5.0 (http://www.jcog.jp/doctor/tool/CTCAEv5J_20220901_v25_1.pdf), the patients with hypothyroidism were classified as follows: 57 patients with grade 1, 54 patients with grades 2-4, 305 patients with normal thyroid function (NTF), and 3 patients with central hypothyroidism.

### Diagnosis of ICI-induced hypothyroidism

Thyroid hormone levels were measured in 419 subjects in all participants. The method of measuring thyroid hormones is as previously described (4). All blood tests after ICI administration were checked and classified as NTF if the TSH was within the institutional reference range. When there was a transient or permanent increase in TSH to 4.12-10.0 μIU/mL and FT4 was not high at that time, the patient was classified as CTCAE grade 1; when TSH was above 10.0 μIU/mL and FT4 below 0.68 ng/mL, or when levothyroxine replacement therapy was started due to hypothyroidism, the patient was classified as CTCAE grade 2-4. Transient thyrotoxicosis was defined as TSH less than measurement sensitivity and FT4 > 1.26 ng/mL that improved spontaneously without antithyroid therapy. The study included 32 participants with transient thyrotoxicosis; 24 participants had Graves’ disease ruled out by thyroid-stimulating antibodies or by evaluation with thyroid ultrasound. None of the participants in this study presented with permanent thyrotoxicosis.

### Statistical analysis

Data are expressed as mean and standard deviation. The primary endpoint was to assess the correlation between the severity of thyroid-related irAEs and the efficacy of immunotherapy. The secondary endpoint was to determine the clinical characteristics of thyroid-related irAEs. The prevalence of thyroid-related autoantibodies in NTF and grade 1 and grades 2-4, the remission rate by RECIST (Response Evaluation Criteria in Solid Tumors) criteria, and the prevalence of malignancy by stage were evaluated with a chi-square test. The frequency of thyroid-related irAEs by each ICI was also used as a chi-square test. Differences in thyroid hormone levels between patients with and without thyroid-related irAEs were evaluated using ANOVA and the Tukey method as a *post hoc* test. To assess one-year survival by the presence of thyroid-related irAE, patients were divided into groups by the presence or absence of thyroid-related irAE within one year after ICI administration, and calculated by chi-square test for survival or death at one year after ICI administration. The mean observation period with and without thyroid-related irAEs during the study period was calculated using nominal logistic analysis adjusted for age, sex, stage of cancer, and ICI used. The observation period was calculated as the number of days alive from the date of the first ICI administration to March 31, 2023. Patients who were transferred during the treatment was calculated as survivors, with the observation period censored at the date of the last visit. When the date of death was known even after the patient was transferred to a hospital, the date of death was used as the end date of observation. JMP version 16.0.2 (SAS Institute Inc. North Carolina. USA) was used for all statistical analysis, and Microsoft Excel for Mac version 16.71 (Microsoft Co. WA. USA) was used to create tables.

## Results

### Clinical characteristics of the participants in this study

**Figure 2:**
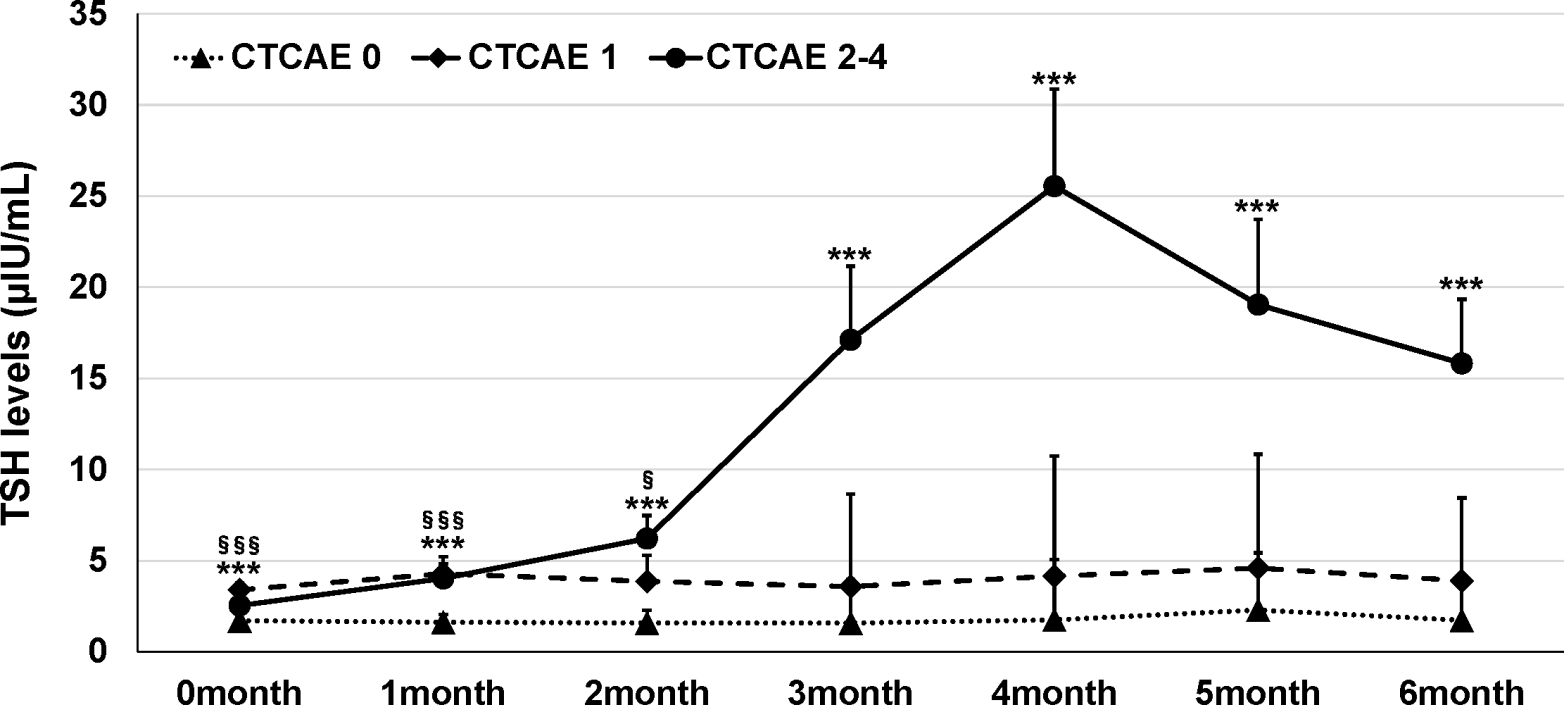
Trends in TSH levels in this study participants. Dots indicate mean values; error bars indicate 95% CI. ANOVA was used to compare with NTF. ***p < 0.0005 (NTF vs Grade 2-4). §p < 0.05, §§§p < 0.0005 (NTF vs Grade 1).

The mean age of the study participants was 68.2 ± 10.3 years, and the percentage of male participants was 72.6%. There was no significant difference between participants with abnormal thyroid function and NTF (p=0.48, 0.56, respectively). ICIs were administered 1.4 ± 3.8 times in NTF, 2.6 ± 5.3 times in Grade 1, and 3.5 ± 4.9 times in Grades 2-4, with a significantly higher number of times in Grades 2-4 compared to NTF (p=0.0024). The frequency of thyroid dysfunction by ICI and the primary disease of the participants are shown in **Table 1**. The frequency of thyroid dysfunction in the study participants was 28.2%. The incidence induced by the drug was 54.2% for ipilimumab/PD-1 inhibitor combination therapy, 26.7% for PD-1 inhibitors, and 26.0% for PD-L1 inhibitors, with the highest incidence in participants receiving combination therapy (p = 0.014). There was a significant difference in the proportion of primary disease for which each ICI was administered (p<0.0001). **Table 2** shows the treatment details at the time of ICI administration for participants who developed thyroid-related irAE. Of the participants who developed Grade 2-4 hypothyroidism, 61.1% were treated with ICI alone, and 38.9% were also treated with some other anticancer agent besides ICI at the same time. Trends of TSH levels of NTF, grade 1, and grades 2-4 for six months after ICI administration are shown in **Figure 2**. TSH levels were significantly higher in Grade 1 and Grades 2-4 when treated with ICI compared to NTF (p<0.0001, p<0.0001, respectively). Transient thyrotoxicosis occurred in 8 patients (2.6%) with NTF, 4 patients (7.0%) with grade 1, and 20 patients (37.0%) with grades 2-4. The mean time from ICI administration to the diagnosis of irAE was 215.9 (95%CI: 153.2-278.7) days for grade 1, and 165.6 (95%CI: 102.9-228.4) days for grades 2-4. In grades 2-4, levothyroxine was administered to 47 patients (82.5%). The positive rates of TgAb and TPOAb in each group are shown in **Table 3**. The positive rates of TgAb and TPOAb increased in line with the increase in CTCAE grade, with transient thyrotoxicosis having the highest positive rate (p<0.0001). The mean dose of levothyroxine was 67.1 μg/day (95% CI: 0-200). Four of the 47 patients were able to stop treatment with levothyroxine, and the time to stop was 385.5 days (95%CI: 87-712).

**Table 1:** Prevalence of endocrine-related immune-related adverse events after treatment with various immune checkpoint inhibitors and percentage of primary disease.

	Ipilimumab and PD-1 inhibitors (n=24)	Nivolumab (n=165)	Pembrolizumab (n=153)	Atezolizumab (n=61)	Dulbumumab (n=12)	Avelumab (n=4)
Any thyroid dysfunction (%)	54.1	26.7	24.1	26.2	16.7	50.0
Grade 1 (%)	16.7	17.0	9.8	14.8	0	25.0
Grades 2-4 (%)	33.3	9.7	13.1	11.5	16.7	25.0
Central hypothyroidism (%)	4.2	0	1.3	0	0	0
percentage of primary disease						
Primary lung cancer (%)	16.7	23.6	50.3	52.5	100	0
Gastrointestinal Tumors (%)	0	49.7	6.5	0	0	0
Renal and urinary tract tumors (%)	37.5	3.5	25.5	0	0	100
Hepatocellular carcinoma (%)	0	0	0	42.6	0	0
Malignant melanoma (%)	37.5	6.7	6.5	0	0	0
Otolaryngological tumors (%)	4.2	7.9	5.9	0	0	0
Other tumors (%)	4.2	5.5	3.9	4.9	0	0
Cancer of unknown primary (%)	0	0	3.0	0	0	0

PD-1; programmed cell death protein 1, PD-L1; programmed cell death protein 1 ligand 1. There was significant difference in the prevalence of abnormal thyroid function in subjects treated with each ICI (chi-square test) (p < 0.0001).

**Table 2:** Anticancer drug concomitantly used from ICI administration to the time of thyroid-related irAE.

Anticancer drug	Grade 2-4
ICI only (%)	61.1
Bevacizumab (%)	11.1
Axitinb (%)	9.3
Carboplatin, pemetrexed (%)	7.4
Cabozantinib (%)	5.6
Lenvatinib (%)	3.7
5-Fluorouracil (%)	1.9
Anticancer drug	Grade 1
ICI only (%)	70.2
Bevacizumab (%)	8.8
Carboplatin, etoposide (%)	7.0
Carboplatin, pemetrexed (%)	5.3
Carboplatin, paclitaxel (%)	5.3
Axitinb (%)	1.8
Ramucirumab (%)	1.8

ICI, immune checkpoint inhibitor.

**Table 3:** Positivity rates for TgAb and TPOAb among the participants in this study.

	TgAb positive	TPOAb positive
NTF (%)	0	0
Grade 1 (%)	8.3	15.4
Grades 2-4 (%)	41.4	26.7
Transient thyrotoxicosis (%)	58.8	31.6

TgAb; anti-thyroglobulin antibody, TPOAb; anti-thyroid peroxidase antibody, NTF; normal thyroid function. There was significant difference in positivity rates for TgAb and TPOAb among the subjects with NTF, Grade 1, Grades 2-4 and transient thyrotoxicosis (chi-square test) (p<0.0001, <0.0001, respectively).

### Thyroid dysfunction and survival ratio after ICI administration

**Figure 3:**
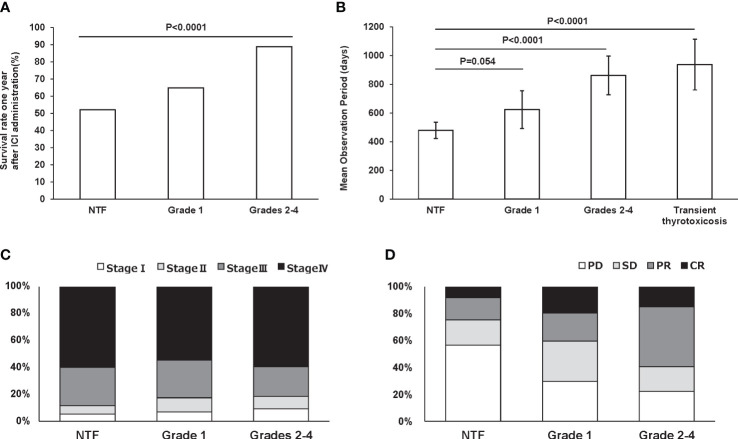
One-year survival rate in subjects with NTF (n = 329), grade 1 (n = 43), and grades 2-4 (n = 47) within one year after ICI administration **(A)**. Mean observation period for each group adjusted for age, gender, ICI used, and stage of cancer. Error bars indicate 95% CI **(B)**. Percentage of malignancy stage **(C)** and RECIST criteria **(D)** in each group.

**Figure 3A** shows the survival rates for each group within 1 year after ICI administration: 43 patients had a grade 1 thyroid-related irAE and 47 patients had a grade 2-4 thyroid-related irAE within 1 year after ICI administration. The 1-year survival rates for participants who developed grade 1 and grade 2-4 hypothyroidism within 1 year after ICI administration were 64.9% and 88.9%, respectively. On the other hand, the 1-year survival rate for participants who had NTF within 1 year after ICI administration was 52.1%, with a significantly longer observation period in those who developed thyroid disorders (p<0.0001). Next, the mean observation period in the participants during the entire post-ICI observation period was significantly longer: grade 2-4 861.8 (95%CI: 726.9-996.7) days and transient thyrotoxicosis 937.8 (95%CI: 760.6-1114.9) days compared to NTF 479.2 (95%CI: 422.5-536.0) days (p<0.0001, <0.0001, respectively) (**Figure 3B**). Cancer stage at the time of ICI administration did not differ among the groups (p=0.68) (**Figure 3C**). Nevertheless, the remission rate assessed by RECIST criteria was significantly higher in grades 2-4 compared to NTF (p<0.0001) (**Figure 3D**).

### Differences in thyroid-related irAE and 1-year survival by gender

**Figure 4:**
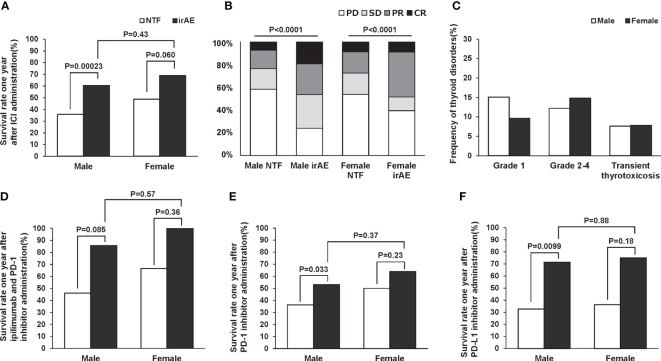
Survival rate one year **(A)** and remission rate **(B)** after ICI administration by gender according to the presence or absence of thyroid-related irAEs. Frequency of thyroid disorders by sex **(C)**. Gender difference in 1-year survival with and without thyroid-related irAEs for each ICI; ipilimumab and PD-1 inhibitors **(D)**, PD-1 inhibitors **(E)**, and PD-L1 inhibitors **(F)**.

Past studies have reported possible gender differences in the incidence of irAE (6). Therefore, we evaluated the difference in one-year survival rate and treatment efficacy by gender. In the subjects of this study, there was a trend toward a higher survival rate after one year of ICI administration in both male and female patients with thyroid-related irAE (**Figure 4A**). And in both men and women, there was a trend toward significantly higher remission rates after ICI administration in participants with thyroid-related irAE (**Figure 4B**). Disease frequencies by gender and ICI used are shown in **Table 4**. There was a significant difference in disease frequency between male and female who received each ICI (ipilimumab and PD-1 inhibitors: p=0.032, PD-1 inhibitors: 0.071, PD-L1 inhibitors: 0,0038). Next, we evaluated the differences in the severity of thyroid disorders according to gender, and the results are shown in **Figure 4C**. There was no gender difference in the frequency of Grade 1, Grade 2-4, and transient thyrotoxicosis (p=0.20). Survival rates one year after ICI administration at each ICI are shown in **Figures 4D–F**. Male with thyroid-related irAE tended to have significantly higher survival rates than subjects without irAE in any ICI. In female, survival was higher for those with thyroid-related irAE compared to subjects without irAE, although this difference was not statistically significant. There was no gender difference in survival for subjects with the appearance of thyroid-related irAE.

**Table 4:** Percentage of disease by gender and drug among participants in the study.

	All subjects (n=419)		Ipilimumab and PD-1 inhibitors (n=24)		PD-1 inhibitors (n=318)		PD-L1 inhibitors (n=77)	
	Male (n=304)	Female (n=115)	Male (n=19)	Female (n=5)	Male (n=223)	Female (n=95)	Male (n=62)	Female (n=15)
Primary lung cancer (%)	42.1	31.3	21.1	0	38.6	31.6	61.3	40.0
Gastrointestinal Tumors (%)	21.1	24.4	0	0	28.7	29.5	0	0
Renal and urinary tract tumors (%)	14.8	11.3	47.4	0	14.8	12.6	4.8	6.7
Hepatocellular carcinoma (%)	6.9	4.4	0	0	0	0	33.9	33.3
Malignant melanoma (%)	4.6	13.9	21.1	100	4.5	11.6	0	0
Otolaryngological tumors (%)	6.3	3.5	5.3	0	4.2	4.2	0	0
Other tumors (%)	2.6	9.6	5.3	0	3.1	8.4	0	20.0
Cancer of unknown primary (%)	1.6	1.7	0	0	4.5	2.1	0	0

PD-1; programmed cell death protein 1, PD-L1; programmed cell death protein 1 ligand 1.

## Discussion

These retrospective data showed that survival was significantly higher in patients who developed abnormal thyroid function after treatment with ICI. It was also found that the greater the degree of thyroid dysfunction, the higher the remission rate with ICI treatment. The data in this study clarified the characteristics of thyroid-related irAEs caused by ICI administration in Japanese patients.

ICIs cause a variety of irAEs, and in the endocrine field thyroid dysfunction is most frequently observed (7). Almost all cases with ICI-induced thyroid dysfunction suffer from destructive thyroiditis or hypothyroidism (8), and hyperthyroidism is rarely observed (9). In this study with Japanese subjects, none of the patients developed permanent hyperthyroidism, and many cases who developed transient thyrotoxicosis progressed to hypothyroidism. What was interesting in this study was that the greater the degree of thyroid dysfunction, the greater the remission rate with ICI. Even grade 1 hypothyroidism, which does not require treatment with levothyroxine, had a higher remission rate than NTF. The same was true for transient hypothyroidism. Previous meta-analyses showed more anti-tumor effects and progression-free survival in patients with thyroid-related irAE (10). It was reported that in Australians with malignant melanoma there was no significant correlation between thyroid-related irAE and treatment remission rates (4). The present report is a valuable analysis of Japanese patients with any carcinoma treated with ICIs, showing not only improved survival but also a trend toward higher remission rates after ICI treatment in participants together with ICI-induced hypothyroidism.

ICI-induced hypothyroidism is most common after CTLA-4 inhibitor/PD-1 inhibitor combination therapy, with PD-1/PD-L1 inhibitors often being the next most frequent. A similar trend was observed in the participants of this study (11). On the other hand, the frequency of hypothyroidism after treatment with ICI varies from study to study depending on the definition of the disease. In this study, primary hypothyroidism was objectively evaluated using CTCAE, and it was found that 28.2% of patients who received ICIs developed some form of thyroid dysfunction. Treatment of hypothyroidism requires the administration of levothyroxine, often with permanent supplementation (12). In this study, 84% of patients with grades 2-4 also required levothyroxine replacement therapy for an average of 167 days after ICI administration. The final dose of levothyroxine was 69.0 μg/day (95% CI: 0-200). After starting ICIs, thyroid function tests should be performed regularly to carefully monitor thyroid hormone levels (13). In addition, the greater the degree of thyroid disorder, the higher the positive rate of thyroid-associated autoantibodies in the participants in this study. It has been reported that the presence of thyroid-associated autoantibodies at the time of ICI administration is significantly correlated with grade 2 or higher irAE. Therefore, the measurement of thyroid-associated autoantibodies at the time of ICI administration may be very important from the clinical point of view (14, 15).

There are several limitations in this study. First, this is a single-center retrospective observational study. All participants in this study were Japanese, and the results of this study may not reflect the situation in other countries due to racial differences and differences in treatment strategies in other countries. Second, 38.9% of the patients who developed hypothyroidism in this study were taking other concomitant anticancer drugs, and thus we failed to exclude the possibility that drug-induced hypothyroidism was caused by these other drugs. Third, this study analyzed information based on medical records, and in many cases, it was not possible to identify the trigger that led to the initiation of levothyroxine replacement therapy. Therefore, it was difficult to group the CTCAE grade 2-4 patients in detail. In addition, only 24 of the 32 participants diagnosed with transient thyrotoxicosis were tested for Graves’ disease, and 8 participants were not adequately differentiated for Graves’ disease. Also, there was no significant difference in survival rates among female subjects with or without thyroid-related irAE in this study. This result may be due to the small number of female subjects compared to male subjects and differences in the frequency of tumor incidence. Future analysis of sex differences in tumor and drug treatment efficacy should be conducted with an alignment of background factors. Finally, patients whose ICI treatment was interrupted or who presented with non-thyroidal irAE may have been included in the NTF, but this point was not considered in this study.

In conclusion, ICI-induced thyroid dysfunction was significantly correlated with survival and mean observation period, and treatment remission rate. These data clearly indicated that the appearance of thyroid dysfunction after ICI treatment would allow us to infer a favorable treatment effect, although there seemed to be some difference among races and regions in the correlation between the appearance of abnormal thyroid function and ICI treatment remission rate.

## Data availability statement

The original contributions presented in the study are included in the article/supplementary material. Further inquiries can be directed to the corresponding author.

## Ethics statement

The studies involving human participants were reviewed and approved by the Institutional Review Board of Kawasaki Medical School. Written informed consent for participation was not required for this study in accordance with the national legislation and the institutional requirements.

## Author contributions

YI designed the study. YI, TK, KD, MO, HT, HI, JS, YF, YK, MS, SN, TM, and HK treated patients and collected data. YI analyzed the data. YI, TK, MS, SN, TM and HK contributed to discussion. KK supervised the project. YI wrote the manuscript. HK reviewed and edited the manuscript.
